# Effects of maternal antenatal treatment with two doses of azithromycin added to monthly sulfadoxine-pyrimethamine for the prevention of low birth weight in Burkina Faso: an open-label randomized controlled trial

**DOI:** 10.1186/s12936-023-04530-5

**Published:** 2023-03-17

**Authors:** Moussa Lingani, Serge H. Zango, Innocent Valéa, Sékou Samadoulougou, Georges Somé, Maïmouna Sanou, Berenger Kaboré, Toussaint Rouamba, Herman Sorgho, Marc C. Tahita, Karim Derra, Michèle Dramaix, Halidou Tinto, Philippe Donnen, Annie Robert

**Affiliations:** 1 Institut de Recherche en Sciences de La Santé/Direction Régionale du Centre Ouest (IRSS/DRCO), BP 18, Nanoro, Burkina Faso; 2 École de Santé Publique, Université Libre de Bruxelles. CP594, Route de Lennik 808, 1070 Brussels, Belgique; 3 Evaluation Platform On Obesity Prevention, Quebec Heart and Lung Institute Research Center, Quebec City, QC G1V 4G5 Canada; 4 Epidemiology and Biostatistics Research Division, Institut de Recherche Expérimentale Et Clinique, Université Catholique de Louvain, Brussels B1.30.13, Clos Chapelle-Aux-Champs 30, B-1200 Brussels, Belgique

**Keywords:** Low birth weight, Miscarriage, Stillbirth, Preterm birth, Sulfadoxine-pyrimethamine, Azithromycin, Burkina Faso

## Abstract

**Background:**

Exposure during pregnancy to malaria and sexually-transmitted infections is associated with adverse birth outcomes including low birth weight (LBW). This study aimed at assessing if the adjunction of two doses of azithromycin to sulfadoxine-pyrimethamine for the intermittent preventive treatment of malaria in pregnancy can reduce LBW.

**Methods:**

A two parallel-groups, open-label randomized controlled trial involving pregnant women (16 to 35 years of age and 12 to 24 weeks of gestation as confirmed by last menstrual period or fundal height) was conducted in rural Burkina Faso. Women were assigned in a 1:1 ratio either to use azithromycin (1 g daily for 2 days) during the second and third trimesters of pregnancy plus monthly sulfadoxine-pyrimethamine (1500/75 mg) (SPAZ) (intervention) or to continue using a monthly sulfadoxine-pyrimethamine (1500/75 mg) (SP) (control). Primary outcome was a LBW (birth weight measured within 24 h after birth < 2500 g). Secondary outcomes including stillbirth, preterm birth or miscarriage are reported together with safety data.

**Results:**

A total of 992 pregnant women underwent randomization (496 per group) and 898 (90.5%) valid birth weights were available (450 in SPAZ and 448 in SP). LBW incidence was 8.7% (39/450) in SPAZ and 9.4% (42/448) in controls (p-value = 0.79). Compared with controls, pregnant women with SPAZ showed a risk ratio (RR) of 1.16 (95% confidence interval (CI 0.64–2.08]) for preterm births, 0.75 (95% CI 0.17–3.35) for miscarriage and 0.64 (95% CI 0.25–1.64) for stillbirths. No treatment-related serious adverse events (SAEs) have been observed, and there was no significant difference in the number of SAEs (13.5% [67/496] in SPAZ, 16.7% [83/496] in SP, p-value = 0.18) or AEs (17.1% [85/496] in SPAZ, 18.8% [93/496] in SP, p-value = 0.56).

**Conclusion:**

Adequate prevention regimen with monthly sulfadoxine-pyrimethamine given to all pregnant women has been proved to reduce the risk of LBW in malaria endemic areas. Adding azithromycin to the regimen does not offer further benefits, as far as women receive a malaria prevention regimen early enough during pregnancy.

*Trial registration* Pan African Clinical Trial Registry (https://pactr.samrc.ac.za/Search.aspx): PACTR201808177464681. Registered 21 August 2018.

## Background

Low birth weight (LBW, < 2500 g) caused by impaired fetal growth or preterm birth (PTB, < 37 weeks) is the biggest driver of neonatal mortality [1]. The global prevalence of LBW is unevenly distributed and sub-Saharan Africa (SSA) is disproportionately affected with nearly 14% of all infants born with a low birth weight [2]. In this region, LBW contributes for more than 80% of the 1,000,000 neonatal deaths that occur each year and it also increases the risk of post-neonatal mortality [1, 3]. In SSA, malaria in pregnancy (MiP) is associated with 900,000 LBW each year, mainly by intrauterine growth restriction [4, 5], and intrauterine infections and their induced inflammatory responses contribute for more than 20% of LBW occurrence [6]. These infections are endemic in SSA [7, 8] and are usually asymptomatic in pregnancy with high risk of misdiagnosis and missed treatment [9, 10].

The intermittent preventive treatment of malaria in pregnancy (IPTp), is a World Health Organization (WHO) recommended strategy required to prevent malaria during pregnancy in endemic countries and consists of a periodic administration of sulfadoxine-pyrimethamine to all pregnant women which clears asymptomatic infections and prevent from new infections [11]. For intrauterine infections, there is no chemoprevention strategy as in malaria, and their management in pregnancy is based on a symptomatic approach [12]. Because these infections are in majority asymptomatic in pregnancy, the utility of the syndrome-based approach is very limited and other approaches are needed if we aim to achieve the sustainable development goal 3.2- to end preventable deaths of newborns and children under 5 years of age [13]. Azithromycin (AZ) is an azalide antibiotic of the macrolides group with a favorable profile for the majority of bacterial pathogens involved in intrauterine infections including gonorrhea, chlamydia and syphilis [14]. This drug can be used as first-line treatment against *Chlamydia trachomatis* [15], *Treponema pallidum* [16, 17], *Neisseria gonorrhea* [18] and, other bacteria related to preterm births [19]. Combining azithromycin to sulfadoxine-pyrimethamine could potentially exhibit a double action against malaria and bacterial infections impact on low birth weight occurrence. To date, the WHO recommends the use of IPTp with sulfadoxine-pyrimethamine for pregnant women in SSA [11], but no chemoprevention is recommended for sexually transmitted infection (STIs). This study aimed to evaluate the efficacy and safety of adding two oral doses of azithromycin to the monthly sulfadoxine-pyrimethamine intermittent preventive treatment of malaria in pregnancy (IPTp-SP) for the prevention of LBW and other adverse birth outcomes including preterm birth, stillbirth and miscarriage in rural areas of Burkina Faso.

## Methods

### Trial design and setting

A two-groups, open-label randomized control trial to compare the use of azithromycin versus nothing in pregnant women receiving monthly sulfadoxine-pyrimethamine for the intermittent preventive treatment of malaria in pregnancy (IPTp-SP), with a 1:1 allocation ratio was conducted. Pregnant women included in the intervention group received monthly sulfadoxine-pyrimethamine (1500/75 mg) and two grams of azithromycin (1 g daily for 2 days) given at the second and third trimesters of pregnancy (SPAZ). Those included in the control group received monthly sulfadoxine-pyrimethamine (1500/75 mg) (IPTp-SP). Azithromycin was administered during the first antenatal visit of the second and third trimesters of pregnancy simultaneously with SP under supervision of study investigators.

The study was carried out in three peripheral health centres of the Yako health district (YHD), in northern Burkina Faso. This region covers a population of 424,577 inhabitants, and where 23,000 pregnancies were exposed to malaria in 2017 [20]. Malaria peaks during the rainy season and represented the main cause of death particularly among pregnant women and children aged less than 5 years [20].

### Participants selection criteria and sample size

Inclusion criteria were age of 16–35 years derived from date of birth, a gestational age of 12–24 weeks assessed through the last menstrual period (LMP) or the fundal height measurement whenever the LMP date was unknown, presence of fetal movements, willingness to adhere to the study protocol and signed informed consent. Women with a personal history of drug allergy, or using cotrimoxazole chemoprophylaxis for HIV were excluded. Women were also excluded if they had multiple gestations (twins). A sample size of 870 pregnant women was required in order to have 80% power to detect a reduction in LBW incidence from 10 to 5% at the significance level of 0.05 using an uncorrected chi-square statistics and equal sized groups, assuming a 10% loss rate, the sample size was increased to 958 pregnant women.

### Screening, enrollment, randomization and follow-up procedures

Pregnant women attending antenatal clinics in three peripheral health centres of the Yako health district catchment areas were invited to participate. Data at screening were extracted from the mothers’ ANC books or by the mother interview when the information was not available in the ANC books. Age, gyneco-obstetrical history, SP uptake, literacy (ability or not to read and write), occupation (having or not an income-generating activity), number of pregnancies (1-primigravida, 2-secondigravida, or ≥ 3-multigravida), and the use of bed nets the night before ANC visit were collected. In addition to physical and obstetrical examinations, blood pressure, axillary temperature, and body weight (during the first ANC visit) were measured, completed by malaria diagnosis in peripheral blood samples by microscopy (positive if any density of asexual malaria parasite), haemoglobin level by spectrophotometry (HemoCue, Ängelholm, Sweden) and body mass index (BMI) [derived by body weight (in kilograms) divided by the square of the height (in metres)].

After baseline characteristics were recorded, eligible pregnant women were randomized at 1:1 ratio to either use azithromycin (1 g daily for 2 days) during the second and third trimesters of pregnancy plus monthly sulfadoxine-pyrimethamine (1500/75 mg) (SPAZ) (intervention) or to continue using a monthly sulfadoxine-pyrimethamine (1500/75 mg) (SP) (control). Each treatment (SPAZ or SP) was randomly assigned to two different treatment codes, (letters A and B). A block randomization procedure was conducted and each block containing four treatment code were prepared by an offsite trial data management team at the CRUN. Allocation codes were kept in consecutively numbered sealed envelopes and revealed after completion of enrolment and immediately prior to treatment. Although, this was not a blinded study in the field and at patient level, other investigators were unaware of the trial group assignment except for the statistician. The intervention consisted of monthly courses of sulfadoxine-pyrimethamine (3 tablets [500/25 mg] given once, Micro Labs Ltd., India) starting early in the second trimester until delivery and azithromycin (2 tablets [500 mg] once daily for 2 days, Pfizer, USA) in the second and third trimesters of pregnancy. The control treatment consisted of monthly sulfadoxine-pyrimethamine (3 tablets [500/25 mg] given once, Micro Labs Ltd., India). Treatment administrations were supervised by a trained nurse or a trained field worker.

### Laboratory procedures for malaria parasitaemia

Thick and thin blood smears were stained with 5% Giemsa for 30 min and independently double-examined by two certified microscopists at 100 × magnification using light microscopy. Thick blood smear was used to detect and count malaria parasites, while thin smear was used to discriminate the *Plasmodium* species that caused the infection. Parasite densities were calculated by counting the number of asexual parasites per 200 white blood cells (WBC), and parasites per μl calculated by assuming a WBC count of 8000 cells per μl of whole blood. When the number of asexual parasites was less than 100 per 200 WBC, counting was done against at least 500 WBC. A slide was declared negative if parasites were not found after a review of 1000 WBC or 100 fields containing at least 10 WBC per field. In case of discrepant results (discrepant species or count difference of at least 50%), a third microscopist assessed the slides. The final result was the average of the two closest readings.

### Outcomes measures

In accordance with the trial protocol, low birth weight (primary outcome) was assessed by measuring birth weight within 24 h after birth by a trained nurse using a calibrated digital infant scale (Seca). Neonates were weighed when unclothed. Duplicate measurements were recorded to the nearest 10 g. If the two measurements differed by 10 g, a third measurement was obtained and the two closest values were averaged. Low birth weight was defined as birth weight of less than 2500 g [21].

Secondary outcomes were preterm birth (a gestation age calculated from the gestational age estimated at the time of inclusion and which is < 37 weeks), stillbirth (birth at ≥ 28 weeks’ gestation with no signs of life), miscarriage (any pregnancy lost at < 28 weeks ‘gestation). Adverse events and serious adverse events were collected throughout the study.

### Statistical analysis

Data was collected on paper-based case report forms (CRF), double-entered in electronic-specific OpenClinica CRF (OpenClinica LLC and collaborators, Waltham, MA, USA, www.OpenClinica.com). All statistical analysis were performed using R software (R Development Core Team, R Foundation for Statistical Computing, Vienna, Austria). Intention-to-treat (ITT) analysis was used and included all women with an available valid birth outcome information. Safety analysis was performed for all women who received a treatment regardless of the number of doses. Proportions between the two groups were compared using the Pearson’s χ2 test. Risk ratios (RR) for prospective studies were calculated for secondary outcomes with their 95% confidence interval. The statistical significance level alpha was set to 0.05.

## Results

### Trial participants

**Fig. 1:**
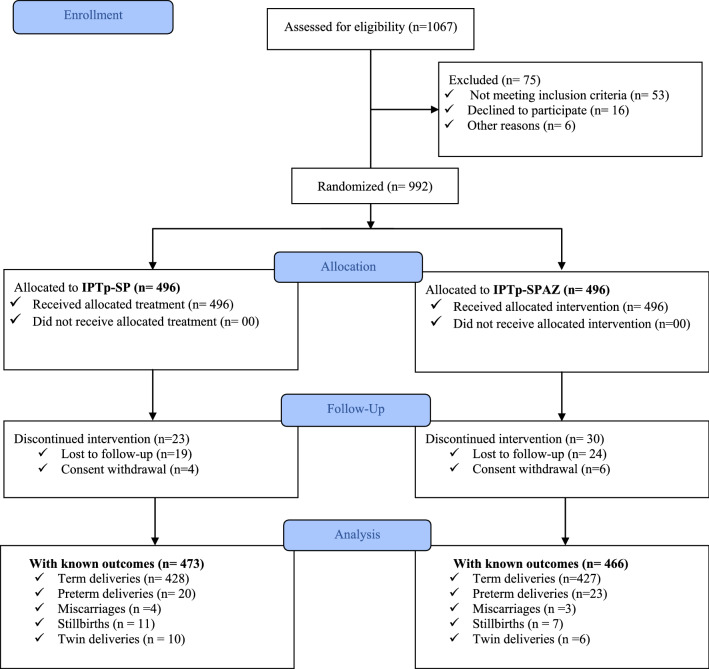
Trial profile

Between August 2019 and June 2021, a total of 992 pregnant women across three health centers underwent randomization, 496 were assigned to the intervention group and 496 to the control group. The 992 pregnancies yielded in 898 neonates birth weight born to women still enrolled in the trial (450 (90.7%) in the intervention group and 448 (90.3%) in the control group) (Fig. 1).

The characteristics of women at baseline were similar in the two groups trial-wide (Table 1). The two groups were thus thought to be comparable according to age, height, body mass index, mean hemoglobin, gravidity, malaria infection, gestational age, fundal height, and sulfadoxine-pyrimethamine uptake. The mean gestational age at baseline was 22.4 weeks in both the intervention group and the control group.

**Table 1:** Baseline characteristics of pregnant women by treatment arm

Characteristics	Intervention arm SPAZ (n = 496)	Control arm SP (n = 496)
Age (years (mean, sd))	26 ± 6	25 ± 6
Body mass index (kg/m^2^ (mean, sd))	23.3 ± 3.5	23.3 ± 3.6
Height (cm, (mean, sd))	163 ± 6	162 ± 6
Fundal height (cm, (mean, sd))	20.5 ± 2.2	20.4 ± 2.4
Gestational age (weeks (mean, sd))	22.4 ± 2.0	22.4 ± 2.0
Hemoglobin at inclusion (g/dL (mean, sd))	10.5 ± 1.3	10.5 ± 1.4
Anemia (Hb < 11 g/dL) (n, %)	219 (60.2)	215 (59.4)
Pregnancy number (n, %)		
Primigravidae (1)	143 (28.8)	149 (30.0)
Secundigravidae (2)	103 (20.8)	121 (24.4)
Multigravida (≥ 3)	250 (50.4)	226 (45.6)
Bed net use the night prior enrolment (n, %)	383 (77.2)	406 (81.9)
Detected parasitemia at inclusion (n, %)	70 (14.6)	84 (17.4)
Literate woman (n, %)	287 (57.9)	288 (58.1)
Having income-generating activities (n, %)	82 (16.6)	98 (19.8)
Maternal history of miscarriage /stillbirth (n, %)	77 (15.5)	80 (16.1)
IPTp-SP uptake before enrolment (n, %)	28 (5.6)	40 (8.1)

*SP* Sulfadoxine-pyrimethamine, *SPAZ* sulfadoxine-pyrimethamine plus azithromycin, *g/dL* gram per deciliter, *cm* centimeter, *kg* kilogram, *m2* square meter, *n* total number per category

### Intervention coverage and adherence

The median number of sulfadoxine-pyrimethamine (SP) doses per woman was 4 (interquartile range between 3 and 5) and the proportion of women with one, two, three, four, or five doses of SP was 0.2% (1/448), 4.9% (2/448), 17.2% (188/448), 42.0% (160/448) and 35.7% (160/448) respectively in the control arm. In the intervention arm, the median number of azithromycin doses was two per pregnant woman and that of SP was 4 (interquartile range between 3 and 5). The proportion of women with one, two, three, four, or five doses of SP in the intervention arm was 0.7% (3/450), 4.2% (19/450), 17.3% (78/450), 38.9% (175/450) and 38.9% (175/450). Overall, 93.3% (418/448) of pregnant women in the intervention arm received the required 4 g of azithromycin.

### Primary and secondary analysis

Among the 992 pregnant women randomized, 939 women (94.7%) remained in the trial with a known birth outcome. Of these, valid birth weights were collected for 914 neonates, data on birth weight represented 97.3% of those with birth outcome information available (456 in the intervention group and 458 in the control group). A total of 16 neonates’ birth weights (6 in the intervention and 10 in the control) were excluded from the analysis due to twin deliveries, thus data for primary outcomes analysis included 898 birth weights (450 in the intervention group and 448 in the control group). The incidence of LBW was 8.7% (39/450) in the intervention group and 9.4% (42/448) in the control group (p = 0.79). Subgroup analyses according to infant sex, the prevalence of malaria infection at baseline, anaemia at baseline did not show a meaningful difference in the intervention effect on the incidence of LBW. For all the secondary outcomes, there was no substantial difference between the intervention group and the control group with respect to the frequency of preterm births, stillbirths and miscarriages (Table 2).

**Table 2:** Low birth weight, preterm birth, miscarriage, and stillbirth by treatment group

Birth outcomes	Intervention arm SPAZ % (n/N)	Control arm SP % (n/N)	RR [95% CI]
Primary outcome			
Low birth weight	8.7% (39/450)	9.4% (42/448)	0.92 [0.61–1.40]
Secondary outcomes			
Preterm birth	5% (23/460)	4.3% (20 /463)	1.16 [0.64–2.08]
Miscarriage	0.7% (3/460))	0.9% (4/463)	0.75 [0.17–3.35]
Stillbirth	1.5% (7/460)	2.4% (11/463)	0.64 [0.25–1.64]

*SP* Sulfadoxine-pyrimethamine, *SPAZ* SP + azithromycin, *RR* Risk ratio, *CI* confident interval

With regard to adverse events, a total of 328 (152 in the intervention group and 176 in the control group) were reported. A total of 67 adverse events in the intervention group and 83 in the control group were classified as serious but none was deemed drug-related (Table 3).

**Table 3:** Safety profile according to treatment groups

Adverse events	Intervention arm SPAZ (N = 496) n (%)	Control arm SP (N = 496) n (%)	p-value^a^
Total events reported	152 (30.6)	176 (35.5)	0.12
Serious AEs	67 (13.5)	83 (16.7)	0.18
Non-serious AEs	85 (17.1)	93 (18.8)	0.56
Detailed AEs			
* Serious events*			
Eclampsia	2 (0.4)	1 (0.2)	0.47
Severe malaria	10 (2.0)	14 (2.8)	0.41
Preterm birth	23 (4.6)	20 (4.0)	0.75
Stillbirth	7 (1.4)	11 (2.2)	0.75
Miscarriage	3 (0.6)	4 (0.8)	0.70
Emergency cesarean section	7 (2.2)	10 (3.6)	0.26
Severe anemia	0 (0.0)	2 (0.4)	0.16
Fetal distress	5 (1.0)	4 (0.8)	0.81
Neonatal death	5 (1.0)	4 (0.8)	0.73
Premature rupture of membrane	3 (0.6)	6 (1.6)	0.13
Neonatal resuscitation	1 (0.2)	3 (0.6)	0.31
Neonatal distress	1 (0.2)	3 (0.6)	0.31
Laparoschisis	0 (0.0)	1 (0.2)	0.32
* Non-serious AEs*			
Threat preterm birth	1 (0.2)	2 (0.2)	0.56
Abdominal pain	10 (2.0)	5 (1.0)	0.20
Cervix infection	0 (0.0)	3 (0.6)	0.08
Endometritis	0 (0.0)	2 (0.4)	0.16
False labor	4 (0.8)	6 (1.2)	0.52
Gastroenteritis	5 (1.0)	8 (1.6)	0.40
Headache	3 (0.6)	5 (1.0)	0.47
Uncomplicated malaria	7 (1.4)	9 (1.8)	0.61
Neonatal infection	4 (0.8)	6 (1.2)	0.52
Oligo-amnios	2 (0.4)	3 (0.2)	0.56
Pelvic pain	5 (1.0)	3 (0.6)	0.48
Pelvic spasm	5 (1.0)	9 (1.8)	0.42
Pneumonia	5 (1.0)	7 (1.4)	0.56
Preeclampsia	5 (1.0)	6 (1.2)	0.76
Urinary infection	1 (0.2)	3 (0.6)	0.18
Others	17 (3.4)	11 (2.2)	0.34

Others included cough, epigastralgia, nausea, oral mycosis, respiratory distress

*AEs* adverse events, *SP* Sulfadoxine-pyrimethamine, *SPAZ* SP + azithromycin

^a^chi-square or fisher exact test used for comparison

## Discussion

In this randomized controlled trial conducted in three peripheral health centres of the Yako health district in rural areas of Burkina Faso, systematically adding azithromycin to sulfadoxine-pyrimethamine during the intermittent preventive treatment of malaria in pregnancy did not result in a significantly lower incidence of low birth weight among neonates than the use of sulfadoxine-pyrimethamine. This was inconsistent with the expectations based on studies suggesting that a systematic combination of anti-malarials and antibiotics to prevent bacterial sexually-transmitted infections and malaria in pregnancy is associated with lower incidence of adverse birth outcomes [8, 22]. Intention to treat analyses of two randomized controlled trials likewise showed no significant impact on low birth weight [23, 24]. In contrast, studies in other areas showed a significant impact of the intervention in the reduction of low birth weight [25, 26].

There are several possible reasons for these finding. First, the overall reduction of the incidence of low birth weight in the control group than that hypothesized to estimate the sample size [27]. Indeed, the intervention was expected to reduce this incidence by half compare to the controls. However, the lower incidence of LBW reported in the control group (9.4%) suggested that progress were made for reducing LBW or that recruited women were less at risk than those entered in epidemiological studies conducted before in the area. Comparing characteristics of women, the clinical trial enrolled pregnant women at an earlier gestational age than pregnant women in epidemiological studies. This finding suggests that adequately and early implementing the sulfadoxine-pyrimethamine intermittent preventive treatment of malaria in pregnancy is efficacious in reducing low birth weight as showed by several studies [28–30]. Second, the efficacy of sulfadoxine-pyrimethamine may not be compromised in west Africa as in most of Eastern African countries. According to a recent study, the high prevalence of *pfdhps540E* mutation was to date restricted to East and South East Africa, which is reassuring for continued use of sulfadoxine-pyrimethamine for the intermittent preventive treatment of malaria in pregnancy in West Africa to reduce adverse birth outcomes, thus the adjunction of an antibiotic does not offer additional benefits [31]. Third, as the intervention aimed to reduce the contribution of sexually transmitted infections to low birth weight [6, 32, 33], the low prevalence of these infections in the study setting (below 4.0%) [34] does not offers a benefit of systematically adding an antibiotic to sulfadoxine-pyrimethamine to prevent low birth weight [15–18, 35]. Fourth but not least, there could be a high level of antimicrobial resistance to azithromycin in the study settings [36–38], that arose first from the high community level use of antibiotics prior to presentation to the hospital in rural Burkina Faso [39], the poor antimicrobial resistance stewardship at hospital level [40, 41], or the mass azithromycin administration to communities during the last decade against trachoma [42]. The combination of these activities may have increased the resistance level to azithromycin, and severely alters its benefits when administered to pregnant women. In addition, the intervention did not reduced the risk of secondary outcomes as the risk of preterm birth, stillbirth and miscarriages were not significantly different between the intervention and the control group although the study was not powered to determine statistical significance for such differences. Although, these findings contrast those found in the literature, the above mentions factors of poor azithromycin impact on low birth weight may be applicable to these adverse birth outcomes.

Despite that the adjunction of azithromycin to sulfadoxine-pyrimethamine did not offer additional benefits to reduce adverse birth outcomes beyond that of the intermittent preventive treatment of malaria in pregnancy using sulfadoxine-pyrimethamine, the intervention presented a good safety profile. There was no drug related serious adverse events and the frequency of adverse events and serious adverse events did not differ across treatment groups as already noted by several studies [25, 26, 32].

Important study limitations are worth noting as the gestational age was assessed using the last menstrual period or the measurement of the fundal height rather than ultrasounds and this may be subject to misclassification of pregnant women. Also, the investigators and the study participants were not blinded to the treatment and this may have led to selection or information bias. However, these bias effects were mitigated as the main endpoint’s measurements were not subject to a large degree of individual interpretation.

## Conclusion

The study showed that adequate use of monthly sulfadoxine-pyrimethamine for the intermittent preventive treatment of malaria in pregnancy given to all pregnant women is likely to reduce the risk of low birth weight in malaria endemic areas. Adding azithromycin to the regimen however does not appear to offer further benefits.

## Data Availability

The dataset analysed during the current study is available from the corresponding author on reasonable request.

## Abbreviations

AE: Adverse events
ANC: Antenatal care
CRUN: Clinical research unit of Nanoro
IPTp: Intermittent preventive treatment of malaria in pregnancy
ITN: Insecticide-treated bed bet
ITT: Intention-to-treat
LBW: Low birth weight
RR: Risk ratio
PTB: Preterm birth
SP: Sulfadoxine-pyrimethamine
AZ: Azithromycin
WHO: World Health Organization
